# Astonishing Impudence

**Published:** 1859-03

**Authors:** 


From the Cincinnati Lancet & Observer.

### *Astonishing Impudence.*

One of the most impudent things perpetrated on the scientific profession for a long time, has been witnessed in this city within the last thirty days. And what, good and unsuspecting reader, do you imagine it is? It is nothing else than that Drs. Newton and Bickley, *Professors in the Eclectic Medical Institute*, of this city, have taken not only the contents of the *Edinburgh Medical and Surgical Journal*, but also the name, issuing it under the title of the *Cincinnati Eclectic and Medical Journal!!* We really could scarcely believe our eyes, when we saw the first number. Only think of it; the names of Symme and Newton, Bennett and Bickley, side by side! Think of it, too, medical men, that this *Eclectic school*, this hot-house of steam, homœopathy, phrenology, "roots and yarbs," conducted in the past by men whose only cry against legitimate medicine has been "poisoners and butchers;" by men who were never heard of beyond the limits of a small country village until their sudden advent before the, public on the foul stage of this so-called school; think of it, we say: of their appropriating, printing and binding up the journal which represents the great Edinburgh University with what they are pleased to call the *Eclectic Journal.*—Faugh! Lanuage fails us to express our disgust. Yet it is a fact.

It is however, to expose these "wolves in sheep's clothing" that we have thus far condescended to notice even so glaring and striking a piece of medical impudence, in these latter days of brilliant humbugs and deceptions. At last, be it known, these pigmy leaders of a dirty and miserable foray on legitimate medicine find that their stock in trade is about giving out, that, like an old prostitute, who has lost her charms, they find it necessary to assume a virtue which they never had, and never will have, solely for the purpose of sustaining a pitiful delusion.

Yes, plain *Eclectic Medical Journal* was proving a bad card. It had ceased any longer to delude; it had failed to establish for this small sect—sprung originally from two remedies. a hot-bath and a lobelia emetic—any position in the regular scientific world; it had failed to make any headway, and the time had come for something to be done. Hence the appropriation of the *Edinburgh Medical Journal.* We shall expect at the end of the year to read the announcement of the course of lectures in the "Eclectic Medical Institute and University of Edinburgh!" Stranger things are happening every day. The "Eclectic Medical Institute and University of Edinburgh of *Cincinnati!*" This reads quite as well, and sounds as sweetly on the ear, as *Cincinnati Eclectic and Edinburgh Medical Journal.*

The school which this Eclectic Journal represents is, we believe, well understood by all intelligent and reading Medical men. It was removed to this city from Worthington, a small village in this State. No sooner was it removed here than its professors commenced a scurrilous abuse of the regular profession for the use of mineral medicines and for surgical operations. When they opened here, they performed no operations; all diseases of a surgical nature being advertised as curable by *Eclectic remedies*, or *Eclectic practice.* When the cholera made its appearance, they advertised in all the daily papers the wonderful power of Eclectic practice to cure all cases of that terrible disease, at the same time loudly proclaiming that "old school practice," as they were pleased to call scientific treatment, saved no cases. They published their cases. No one ever heard of any patients dying under their treatment. In addition, they pronounced in the public papers that all mineral medicines were poisons. They blowed what they called Eclectic practice and themselves on the corners of the streets, and in all public places. Next, we find in the faculty the representatives of all known quack systems: homœ-quack, electro-pathic, electro-biological, *et id omne genus*,—black spirits, green spirits, and blue; with their class made up of unsexed *women*, broken down preachers, sickly school teachers, crazy mechanics, and sugar-loaf headed individuals who had but two ideas—terrible opposition to "mineral practice," and a profound admiration for "Eclectic practice." They graduated a large number from this intellectual set, with one year's attendance on their course of lectures.

More still: This Dr. Robert Newton has been a *professed cancer curer*, and for a long time had *a secret remedy* for its treatment.

And what, now, is their course? Still crying out against legitimate medicine, while using the very remedies they have so long villified and abused as poisons. They have advanced, no doubt, from the reading and *editing* of the works of truly scientific authors. Newton gives mineral medicines: he has even given, as we have been told, that much abused remedy, calomel. He has edited *Symme's Surgery*, in which many wonderful and new ideas from *Eclectic surgical practice* have been introduced. He even admits, now, that he can not cure all cases of cancer; that it will return after it has been once removed. He even finds that Electic practice and remedies fail to take a stone out of the bladder, and resorts to the knife.

Such is a brief history of this school and its most prominent man. It once had a fellow in it by the name of Sanders, who had the impudence to edit *Gregory's Chemistry. O tempora! O mores!* And last, but not least, to cap the climax of this grand Eclectic quack school, it has had the impudence, nay, more, has so far stultified itself as to print and publish the journal of all others which has, and does at present, support legitimate scientific medicine.

They wage a continual warfare against the *code of ethics*, and yet have the greatest disposition to be on friendly terms with the regular profession. The editorial department of the January number of this hybrid journal is full of protestations of friendship. Like all the quacks, they seek a corner of the great mantle of true medicine to cover them. They can fraternize with all sorts of quacks, according to their own account.

Some very respectable professional gentlemen, and even journalists, are ignorant of the position of this class of quacks in this city: Newton's school, and Cleaveland's school—the two electic quack concerns of this city, with whom no regular member of the profession has anything to do. If our space permitted, our sketch would be much longer.

Before we close, let us give our readers the contents of the *Cincinnati Eclectic and Medical Journal.* It is nearly all Edinburgh. 1st: "An Eclectic treatise on the practice of medicine, by R. S. Newton, Professor of Surgery," in which he claims that Eclectics brought forward twenty-five years ago the anti blood letting treatment of disease! With the exception of one other article; on ptyalism, from a Dr. Woodruff, of Pittsburgh, Indiana, all the others are taken from the *Edinburgh Medical and Surgical Journal.* Even, too, they cannot write reviews of books, for with the exception of a notice of Dr. Sanger's book on Prostitution, all the others (and there are several) are from the same source.

We are too happy to be able to inform our readers that these two so-called schools are dying slowly. Their great effort to preserve a so-called position is mainly directed to appropriating the books and journals of seientific men, publishing them as Electic treatises, widely different from old school authors. In proof of this, Dr. J. King brought out an *Eclectic Dispensatory*, so full and glaring with wholesale *plagiarisms* from the *U. S. Dispensatory.* that Wood and Bache, its authors, sued out an injunction against its sale, so that Dr. King was forced to rewrite his Dispensatory. So of Dr. Cleaveland, with his *Medical Dictionary*, whom Dr. Reese, of New York, charged boldly with plagiarizing from him. And last, but not least, Newton and Bickley have appropriated one of the first journals in the world representing "old school medicine." The professors of these two schools have amazed the professional public, and even the unprofessional, with the number and variety of books *written* and issued by them. The truth is, not one of them is celebrated in any speciality of medicine in this city. We hesitated for some time before penning this article. We feel it to be our duty to say what we have said, in our capacity as medical journalists of true scientific medicine. We have not written for our friends and readers in this city, but more particularly for our readers and editorial brethen at a distance. We leave these people, their school and journals to time, which sooner or later will number them in oblivion, where so many worthles systems already lie, "unwept, unhonored and unsung." We are not to be draged in to any discussion. Our pages can be devoted to much more profitable matter.

